# HIV Replication Is Not Controlled by CD8^+^ T Cells during the Acute Phase of the Infection in Humanized Mice

**DOI:** 10.1371/journal.pone.0138420

**Published:** 2015-09-25

**Authors:** Nicolas Y. Petit, Sidonie Lambert-Niclot, Anne-Geneviève Marcelin, Sylvie Garcia, Gilles Marodon

**Affiliations:** 1 Sorbonne Universités, UPMC Univ PARIS 06, INSERM, CNRS, Centre d’Immunologie et des Maladies Infectieuses (CIMI), Paris, France; 2 Sorbonne Universités, UPMC Univ PARIS 06, Institut Pierre Louis d’Epidémiologie et de Santé Publique, Paris, France; 3 Institut Pasteur, Paris, France; University of Pittsburgh Center for Vaccine Research, UNITED STATES

## Abstract

HIV replication follows a well-defined pattern during the acute phase of the infection in humans. After reaching a peak during the first few weeks after infection, viral replication resolves to a set-point thereafter. There are still uncertainties regarding the contribution of CD8^+^ T cells in establishing this set-point. An alternative explanation, supported by *in silico* modeling, would imply that viral replication is limited by the number of available targets for infection, i.e. CD4^+^CCR5^+^ T cells. Here, we used NOD.SCID.gc^-/-^ mice bearing human CD4^+^CCR5^+^ and CD8^+^ T cells derived from CD34^+^ progenitors to investigate the relative contribution of both in viral control after the peak. Using low dose of a CCR5-tropic HIV virus, we observed an increase in viral replication followed by “spontaneous” resolution of the peak, similar to humans. To rule out any possible role for CD8^+^ T cells in viral control, we infected mice in which CD8^+^ T cells had been removed by a depleting antibody. Globally, viral replication was not affected by the absence of CD8^+^ T cells. Strikingly, resolution of the viral peak was equally observed in mice with or without CD8^+^ T cells, showing that CD8^+^ T cells were not involved in viral control in the early phase of the infection. In contrast, a marked and specific loss of CCR5-expressing CD4^+^ T cells was observed in the spleen and in the bone marrow, but not in the blood, of infected animals. Our results strongly suggest that viral replication during the acute phase of the infection in humanized mice is mainly constrained by the number of available targets in lymphoid tissues rather than by CD8^+^ T cells.

## Introduction

Thirty years after the discovery of the Human Immunodeficiency Virus (HIV) as the causative agent of AIDS [1], we still do not have a definitive and undisputed mechanism on how viral replication is controlled (or not). Immune correlates for protection in Elite Controllers (a rare subset of patients that control HIV replication without treatment) are missing, although various studies have linked this peculiar clinical status to either genetic [2,3] or immune [4,5] factors.

Detection of HIV-specific CD8^+^ T cells in humans is indisputable [6], but how those exert their anti-viral effect is not clear. CD8^+^ T cells might be particularly important to suppress viral replication during the acute phase of the infection, where the outcome of the infection is more or less determined [7]. Relevant to that point, it was recently shown that viral reservoirs of SIV that persist under treatment are established even before the viral peak [8]. Earlier studies in monkeys have shown that CD8-depletion led not to higher viral loads at the peak, but to a failure in the resolution of that peak [9]. Although cytolysis of infected cells by CTLs was the obvious mechanism to explain the control of SIV replication, it was later reported that depletion of CD8^+^ T cells did not change the decay rate of productively infected cells with or without antiretroviral therapy, suggesting that CD8^+^ T cells do not control viral replication by cytolysis of infected cells [10,11]. It was also reported that CD8-depletion was associated with increased CD4^+^ T cells proliferation which may significantly impact viral replication [12,13]. Also, it was recently reported that control of SIV in Cynomolgus Macaques is not associated with efficient SIV-specific CD8^+^ T cells [14]. Thus, increased viral loads in the absence of CD8^+^ T cells may have been misinterpreted.

The co-evolution of viral variants escaping the CTL response as the disease progresses is perhaps the best 'marker' in favor of CD8-mediated viral control. The best causative evidence for a CD8-mediated viral control has recently been put forward by Siliciano and coll. which reported that anti-HIV CD8^+^ T cells exert their effect on CD4^+^ T cells carrying latent virus through cytolysis *in vitro* [15], in striking contrast with the SIV studies exposed above. Thus, there are still major gaps in our comprehension of the mechanism by which CD8^+^ T cells control viral replication [16] and it is possible that SIV may differ from HIV in that respect.

A simple mathematical model based on limited target cells available for the virus was sufficient to explain most of the viral dynamics observed in patients during the acute phase of the infection [17,18]. More advanced mathematical modeling incorporating a CTL response have led to the conclusion that early viral replication is dictated both by intrinsic dynamics of viral-target interactions and by the magnitude of the initial immune response [19]. However, these *in silico* predictions have been difficult to assess *in vivo* using HIV infection of human CD4^+^ T cells. Although the role for CD8^+^ T cells in controlling HIV infection is still elusive, for the reasons explained above, it is well established that HIV or SIV infection leads to massive CD4^+^CCR5^+^ T cell deletion in peripheral organs and particularly the gastro-intestinal tract [20,21].

Due to the limitations of SIV-infected monkeys as models for HIV infection, humanized mice (HuMice) bearing human T cells susceptible to HIV infection represent an attractive additional model [22]. A simple way of generating HuMice consists in grafting CD34^+^ hematopoietic progenitors in NOD/SCID.gc^-/-^ (NSG or NOG, depending on the nature of the gc mutation [23]) or RAG-2.gc^-/-^ mice on the BALB/c background (BRG) [24]. Here, we used NSG HuMice to interrogate the respective importance of CD8^+^ T cells vs. deletion of CD4^+^CCR5^+^ T cells in the control of viral replication during the acute phase of the infection.

## Results

### Early features of HIV infection in the blood of NSG HuMice

**Fig 1:**
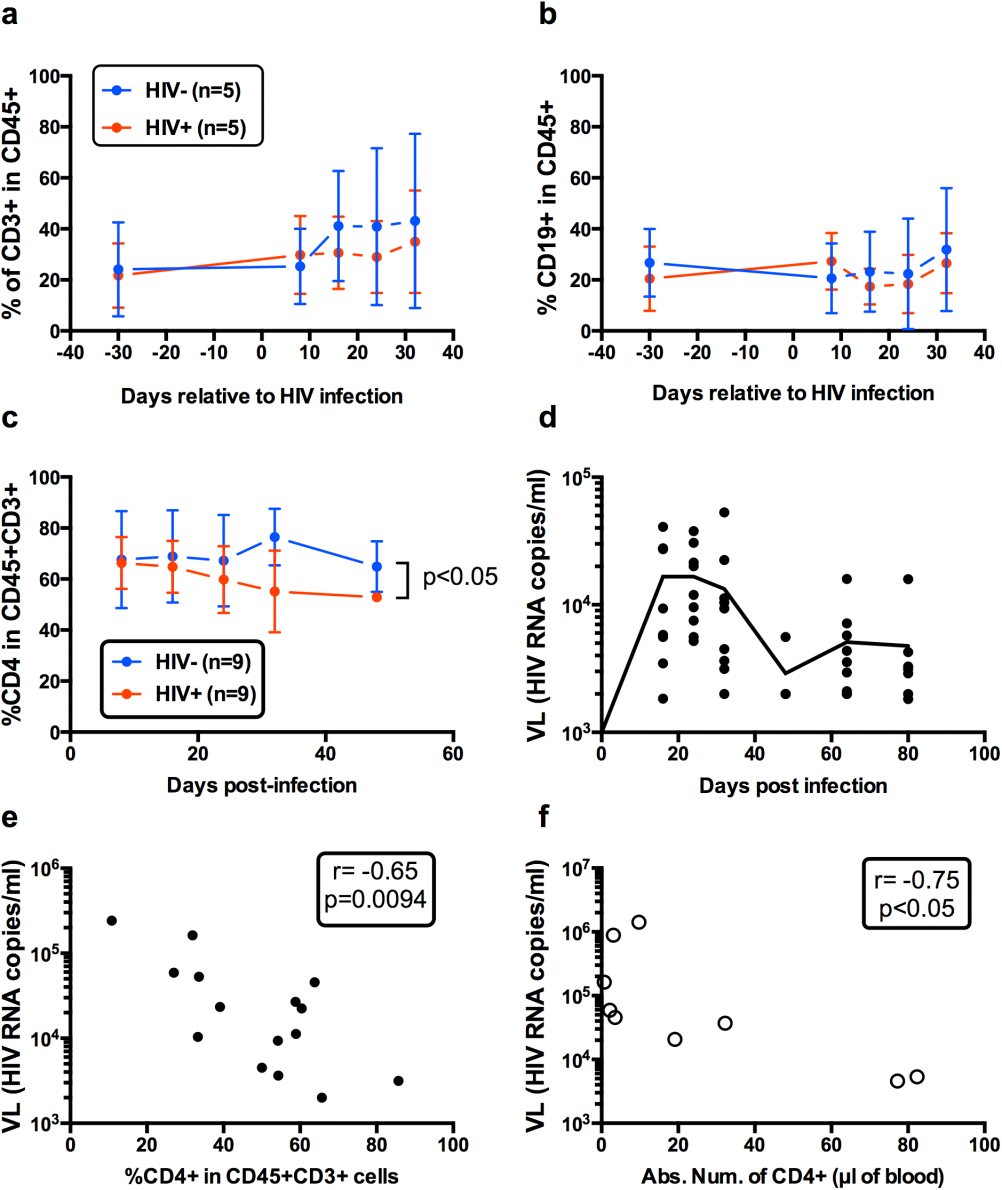
Early features of HIV infection in the blood of NSG HuMice. Frequencies of CD3^+^ T cells (a) and CD19^+^ B cells (b) in CD45^+^ humans cells from the blood of NSG HuMice infected at 19 weeks of age (D0 on the graph) (c) Frequencies of CD4^+^ T cells in CD45^+^CD3^+^ human cells in the blood relative to the day of infection. The p value is from a linear regression analysis. (d) Viral loads (VL) in the blood of HIV-infected NSG HuMice was determined by the Cobas PCR at the time indicated in the legend. Results shown in c and d are from 2 independent experiments. (e) Correlation between viral loads (VL) and frequency of CD4^+^ T cells at 30 days post-infection with 10 to 50 ng p24 of CCR5-tropic HIV. Results shown are cumulative from 4 independent experiments with HuMice aged from 19 to 28 weeks of age when infected. Each dot represents a single mouse. (f) Correlation between viral loads (VL) and absolute numbers of CD4^+^ T cells in the blood of HuMice infected with 50 ng p24 of CCR5-tropic HIV at 25 weeks of age. Results represent cumulative values obtained at 14 and 37 days post infection from a total of 5 mice analyzed in a single experiment. The non-parametric Spearman correlation coefficient and the p value are indicated in panels e and f.

HuMice were generated by neonatal injection of cord blood purified CD34^+^ cells. The kinetics of human hematopoeitic cell reconstitution in the blood was comparable to what we already published in the same model [25]. At the time of infection, there was an equal ratio of T and B cells in CD45^+^ human cells of the blood, which did not significantly change after infection (Fig 1A and 1B and S1 Fig). Thus, representation of human cell subsets was not globally affected by HIV infection during the time frame of this study. Within CD3^+^ T cells, we observed a progressive and significant reduction in the frequencies of CD4^+^ T cells in the HIV-infected group relative to non-infected mice over a period of 50 days (Fig 1C and S1 Fig). A peak viremia was reached between D20 and D30 after infection that was followed by a phase of viral decay to a set-point (Fig 1D). Furthermore, reduced frequencies (Fig 1E) and numbers (Fig 1F) of CD4^+^ T cells in the blood correlated well with increased viral loads. Thus, our results show a peak in viral replication shortly after infection that 'spontaneously' resolved thereafter, accompanied by a loss in CD4^+^ T cells in the blood of infected animals.

### Impact of CD8^+^ T cell depletion on early viral replication

**Fig 2:**
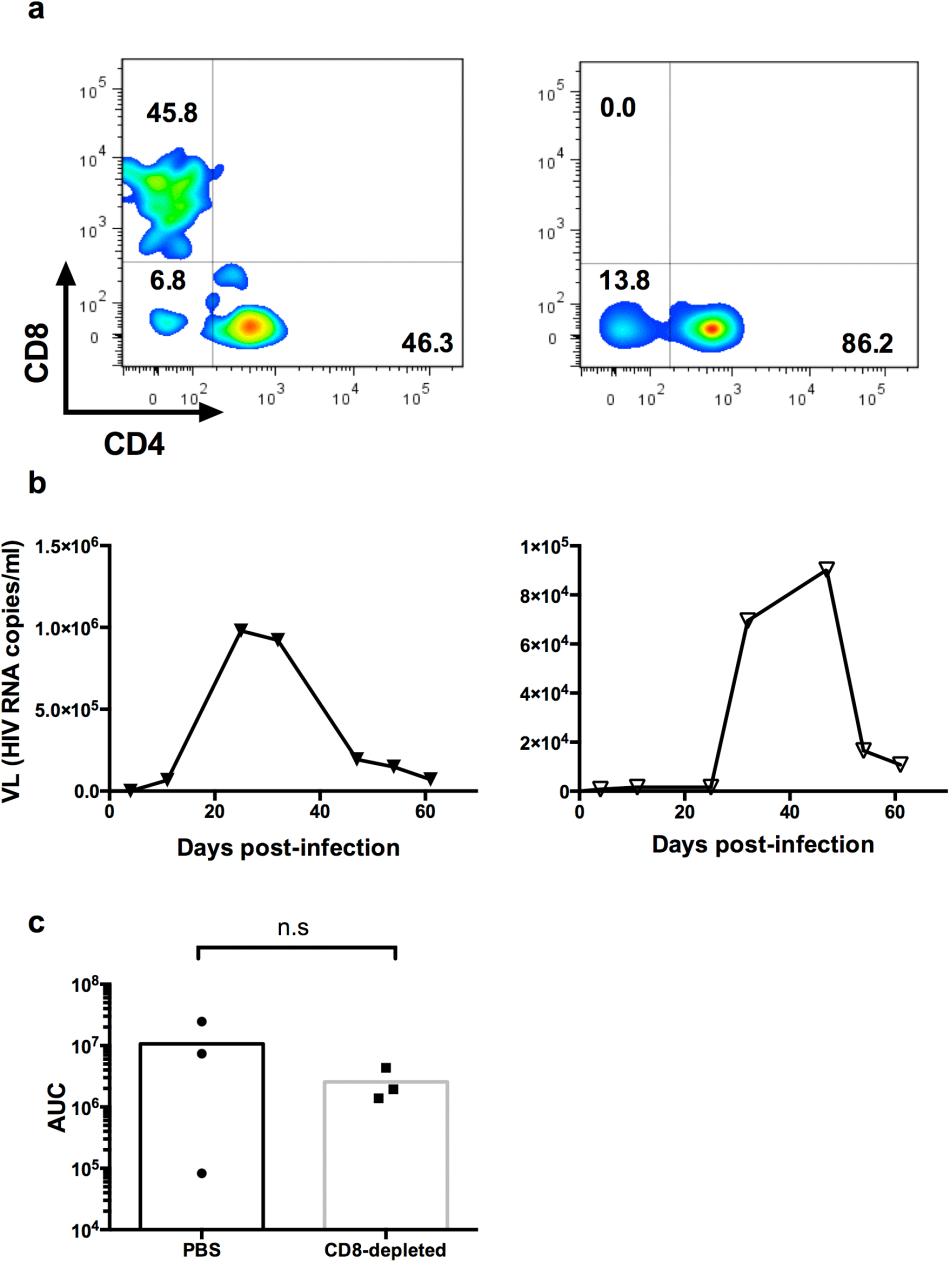
Impact of CD8 T cell depletion on early viral replication in HIV-infected NSG HuMice (a) Representative FACS profile of a CD8/CD4 staining in CD45^+^CD3^+^ cells from the blood 50 days after injection of PBS or 10 mg/kg of a chimeric anti-CD8 depleting antibody (+MT807R1) in 17 weeks-old NSG HuMice. (b) Viral loads (VL) were determined by Cobas Roche PCR in 17 to 36-weeks old NSG HuMice infected with 15 to 25 ng p24 of CCR5-tropic viruses 3 days after injection of PBS or the CD8-depleting antibody (CD8-depleted) (c) Area under the curve (AUC) was determined based on viremia in the blood of HuMice treated with PBS or the CD8-depleting antibody (CD8-depleted) and infected 3 days later with a CCR5-tropic HIV (Bal or NL4A8). (n.s = p>0.05 from a Mann-Whitney test).

According to the dogma, resolution of the viral peak in HuMice would suggest that a functional anti-HIV CD8^+^ T cell-response was responsible for viral control. To test this possibility, we monitored viral replication with or without CD8^+^ T cells, with the expectation of a lower viral control in CD8-depleted animals relative to controls. To deplete human CD8^+^ T cells, we used the improved chimeric anti-simian CD8 MT807R1 [26]. To rule out masking by the MT807R1 Ig for subsequent CD8 staining, we used a clone that still detected the CD8 molecule after short incubation with MT807R1 (S2 Fig). Depletion of CD8^+^ T cells with this reagent was long-lasting since CD8^+^ T cells were still undetected 50 days after injection (Fig 2A). However, viral replication profiles were undistinguishable in terms of kinetics and magnitude in PBS or CD8-depleted animals (Fig 2B and S3 Fig). To accommodate variability amongst animals, the area under the curve (AUC) was determined for each mouse in both groups. The AUC were similar in depleted and non-depleted animals (Fig 2C), showing that CD8^+^ T cells did not globally impact viral replication in HuMice.

### Specific deletion of CD4^+^CCR5^+^ T cells in the periphery but not in the blood

**Fig 3:**
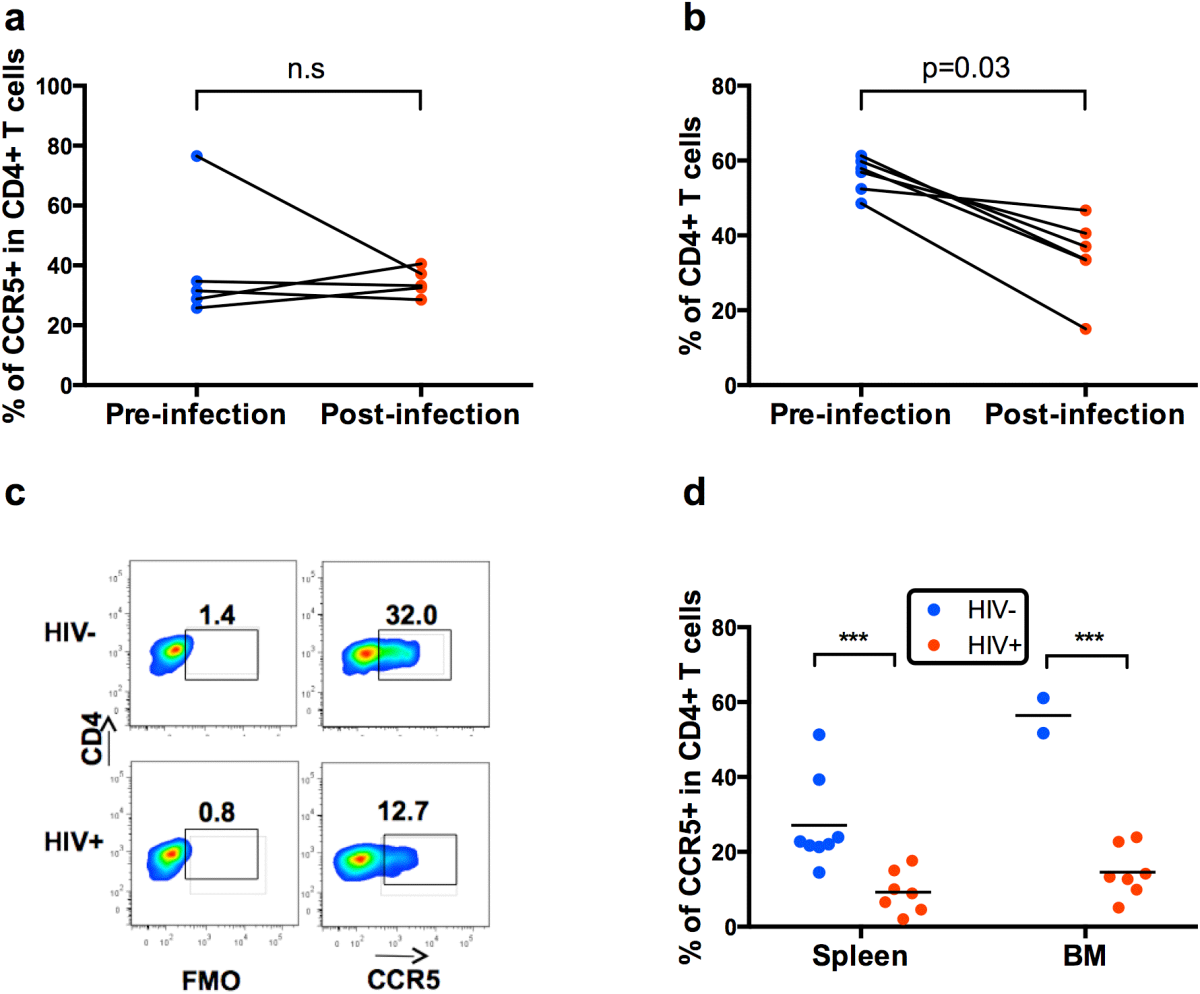
Specific deletion of CD4^+^CCR5^+^ T cells in the periphery but not in the blood. Frequencies of CCR5^+^ in CD45^+^CD3^+^CD4^+^ T cells (a) and of CD4^+^ in CD45^+^CD3^+^ T cells (b) measured in the blood before and 23 days after infection of HIV-infected NSG HuMice (ns = not significant from a Wilcoxon matched-pairs signed rank test) (c) A representative FACS profile showing depletion of CCR5^+^ cells in the spleen of an infected animal is pictured. Numbers indicate the frequency of positive-cells in the depicted gate. FMO = Fluorescence minus one (d) Frequencies of CCR5^+^ cells in human CD45^+^CD3^+^ cells from the spleen and the bone marrow (BM) 37 days after HIV infection (HIV+). In this experiment, 25 weeks-old NSG HuMice were infected with 50 ng p24 of CCCR5-tropic HIV. Control mice (HIV^-^) are non-infected NSG HuMice from two independent experiments euthanized at 13 and 17 weeks of age. Statistical significance was determined using the Holm-Sidak method, with alpha = 5.0%.

We then assessed whether CD4^+^CCR5^+^ T cells, the main target for HIV, would be specifically deleted in HuMice. We first compared the frequencies of CD4^+^CCR5^+^ cells in the blood before and after HIV infection in the same animals. Except for one mice which has a very high frequency of CCR5^+^ cells in CD4^+^ T cells to start with, there was no sign of deletion of this subset 23 days after infection (Fig 3A), even though frequencies of total CD4^+^ T cells were reduced in the same animals (Fig 3B). In contrast, reduced frequencies of CD4^+^CCR5^+^ T cells were readily observed during the same period in the spleen and in the bone marrow if HIV-infected or non-infected HuMice were compared at the end of the experiment 37 days after infection (Fig 3C and 3D). Viral loads at that time were 103,809 ± 61,636 HIV RNA copies/mL. Thus, HIV replication was associated with a loss of CD4^+^CCR5^+^ T cells in lymphoid tissues but not in the blood.

## Discussion

Here we show that depletion of CD8+ T cells did not impact viral replication during the acute phase of the infection in HuMice. In contrast, we observed that CD4^+^CCR5^+^ T cells were reduced in lymphoid tissues. We infer from these results that resolution of the viral peak might be more dependent from the number of target cells than from CD8^+^ T cells, in agreement with *in silico* modeling [19]. To our knowledge, our study and the one from Gorantla et al. [27] represent the only examples directly addressing the role of CD8^+^ T cells in the control of HIV infection *in vivo*. Contrary to our results, Gorantla et al. showed that CD8^+^ T cell depletion during the chronic phase resulted in a modest increase in viral loads. Thus, it is possible that CD8^+^ T cells do not control viral replication during the acute phase whereas they may exert some kind of control during the chronic phase in HuMice. Although Dudek et al. reported the coevolution of HIV and of the CD8^+^ T cell response in BLT mice [28], they did not show that CD8^+^ T cells were responsible for viral control in the model. Indeed, very little viral control was seen even in mice carrying a humanized HLA allele. It would be crucial to determine the impact of depleting CD8^+^ T cells in BLT HuMice.

In studies using related models of HuMice infected with R5-tropic viruses, viremia usually increased up to a plateau and rarely decayed to a viral set-point. Only few studies in HuMice showed the characteristic bell-shaped curved of viremia observed in humans, distancing the model from the physiological reality (reviewed in [22]). Interestingly, even the most advanced model of HIV infection in HuMice failed to mimic the pattern of the primary phase of the infection in humans so far [29], showing that the quality of human cell reconstitution is not responsible for the differences. Although we cannot exclude that our observation is fortuitously similar to the human situation, a human-like pattern of viral replication was also reported by Gorantla et al. in the same NSG HuMice model infected with low dose of the R5-tropic ADA virus [27]. This suggests that NSG HuMice infected with CCR5-tropic HIV might be particularly prone to recapitulate the viral replication pattern observed in humans. One must bear in mind that productive infection in humans is established with an extremely low amount of virus. It has been suggested that up to 40% of IV drug users and as much as 80% of heterosexual transmissions are due to infection by a single virion [30,31]. Therefore, we believe that the key factor to explain why HIV replication in HuMice is reminiscent of the human situation in some but not all studies might be the dose of infectious virus used for infection. Of note, the route of infection does not seem to play a major role since we used intravenous injection whereas Gorantla et al. used the intra-peritoneal route.

Our results confirm that CD4^+^CCR5^+^ cells of the periphery, but not of the blood, are a major target for destruction by HIV [32], like in SIV studies [20,21]. We speculate that fast-moving circulating cells in the blood might not be the ideal target for HIV infection and that slow-moving cells residing in tissues may represent easier targets for HIV. In agreement with that hypothesis, it has been shown in HuMice that migration of infected cells to the lymphoid organs appears necessary for efficient spreading of HIV and syncitia formation [33].

It is well recognized that some human HLA alleles are associated with viral control and it is likely that CD8^+^ T cells play a very important role to control HIV replication in those subsets of patients. Susceptibility to infection is also heavily constrained by host cellular factors, from cell surface molecules, such as CCR5, to intracellular proteins, in which SAMHD1 is only the latest of a long list [34,35]. Non-pathogenic infection of SIV in its natural host African Green Monkeys has also been linked to paucity of CD4^+^CCR5^+^ cells [36]. The Berlin patient represents the spectacular proof-of-evidence that manipulation of CCR5 expression *ex vivo* may lead to a cure [37]. Our results emphasize the need to develop alternative therapeutic strategies independent of the immune response. Such strategies might be based on gene modifications and/or gene transfer, as recently exemplified in a clinical trial using Zinc Finger Nucleases to target CCR5 expression on re-infused T lymphocytes [38]. HuMice might prove invaluable tools in that quest.

## Material and Methods

### Generation of humanized mice and HIV infection

NOD.Cg-Prkdcscid-Il2rgtm1Wjl/SzJ (NSG) mice (stock ≠00557) were bred in animal facilities Centre d’Expérimentation Fonctionnelle (CEF) according to the Jackson Laboratory handling practice specific to that strain (available at www.jax.org). Mice were given 3% fat food and acidified water *ad libitum* and were maintained under a 14-hour light 10-hour dark cycle. Breeding pairs were given Baytril antibiotic every other week. 24hrs-48hrs old newborn NSG mice were irradiated at 1 Gy and grafted with 5.10^4^ to 2.10^5^ human cord blood-purified CD34^+^ cells by the intra-hepatic route. Human hematopoietic progenitor cells were obtained from cord blood samples collected at the Service de Gynécologie Obstétrique-Groupe Hospitalier Pitié-Salpétrière from healthy donors after informed consent. The protocol was approved by the Comité de Protection des Personnes-Ile de France. CD34^+^ progenitors were sorted with human CD34 MicroBeads kit, according to the manufacturer's instructions (Miltenyi). HIV was injected i.v in the retro-orbital sinus in a final volume of 100μl of PBS1x. Mice were infected with viral stocks of HIV Bal, Yu2 or NL-AD8, all strains known to use CCR5 as a co-receptor, with doses indicated on figure legends. Mice were sampled every week for serum by maxillary vein puncture under light anesthesia with Isoflurane. Mice were monitored every other day for signs of sufferings, such as weight loss, hunchback posture or anemia, Mice were euthanized by cervical dislocation. The local ethical committee Darwin approved all mice experimental protocols.

### Viral production and quantification

Plasma HIV-1 RNA viral loads were measured using the COBASw AmpliPrep/COBASw TaqMan HIV-1 Test, version 2.0 (Roche Diagnostics, Branchburg, NJ, USA), with a lower quantification limit of 20 copies/mL. Due to the small volumes of serum from the mice, a dilution was necessary to reach the volume needed for the assay. Thus, this detection limit varied between 200 and 2000 copies/mL depending on the initial volume of mouse serum. Viral stocks were produced by calcium phosphate transfection on 293T cells with 10 μg of plasmids encoding HIV Bal, Yu2 or NL-AD8 (AIDS Reference and Reagent Program, Washington, USA) and were quantified by p24 ELISA according to the manufacturer's instructions (Zeptometrix, Buffalo, NY).

### CD8 depletion

The MT807R1 recombinant Ig was provided by the Nonhuman Primate Reagent Resource (NIH contract HHSN272200900037C and grant RR016001). MT807R1 is a recombinant Ig consisting of rhesus IgG1k constant regions and CDRs derived from the anti-human CD8 antibody M-T807 grafted into rhesus variable framework regions. The antibody was expressed in vitro using serum free medium and purified by protein A affinity chromatography. Endotoxin was <1EU/mg. The MT807R1 Ig was injected i.p at a concentration of 10mg/kg into 17 to 36 weeks-old NSG HuMice. Control mice received the same volume of PBS.

### Flow cytometry

Cell suspensions were stained with optimal quantity of antibodies at a concentration of 10^7^ cells/ml in a final volume of 100μl of PBS/FCS 3%. Incubation was performed in the dark at 6°C for 20 min. The following mAbs were used for cell surface staining: anti-CCR5-APC (2D7; BD Pharmingen), anti-CD4 PerCP (RPA-T4, Biolegend), anti-CD8 A700 or Pacific Blue (HIT8a, RPA-T8, Biolegend), anti-CD8 PE (DK25, DAKO), CD45 APC (HI30, Biolegend), CD3 PeCy7 (UCHT1, Biolegend), CD45 PE-CF594 (HI30, BD Pharmingen). All cell preparations were acquired on an LSRII cytometer (BD) and analyzed with FlowJo software (Tree Star, Portland, OR). Frequencies of positive cells were determined according to the Fluorescence minus one (FMO) staining control. Absolute counts in blood were determined by cell-surface staining in whole blood using TrueCount beads, as recommended by the manufacturer (BD).

## Supporting Information

S1 Fig Human T and B cell reconstitution in NSG HuMice. (a) Frequencies of CD3^+^ T cells and CD19^+^ B cells in CD45^+^ human cells was measured in the blood of 10 NSG HuMice from 14 to 20 weeks of age at the indicated days relative to HIV infection (b) Representative CD4/CD8 profiles from non-infected (HIV-) and infected (HIV+) NSG HuMice at the indicated time after infection with 15ng of HIV Bal at 19 weeks of age.
(PDF)

S2 Fig Masking of CD8 staining by MT807R1 Ig. Resting PBMC were incubated 30 min. on ice with PBS or 1μg of MT807R1. Cells were then washed and stained with fluorescent mAbs anti-CD3, anti-CD4 and various anti-CD8 clones as indicated on the figure. Numbers on the profiles indicate the percentage of CD8^+^ cells.
(PDF)

S3 Fig Viral loads in PBS vs CD8-depleted HIV-infected NSG HuMice. 17 to 36 weeks-old NSG HuMice were injected with PBS or 10mg/kg MT807R1 (CD8-depleted) and infected 3 days later with HIV NLAD8 strain. Viremia was determined using the Cobas Roche amplification PCR.
(PDF)
