# Supplementary figures and images for: HIV Replication Is Not Controlled by CD8+ T Cells during the Acute Phase of the Infection in Humanized Mice
Source: PLoS One. 2015 Sep 25;10(9):e0138420. doi: 10.1371/journal.pone.0138420 (PMC4583499; doi:10.1371/journal.pone.0138420)

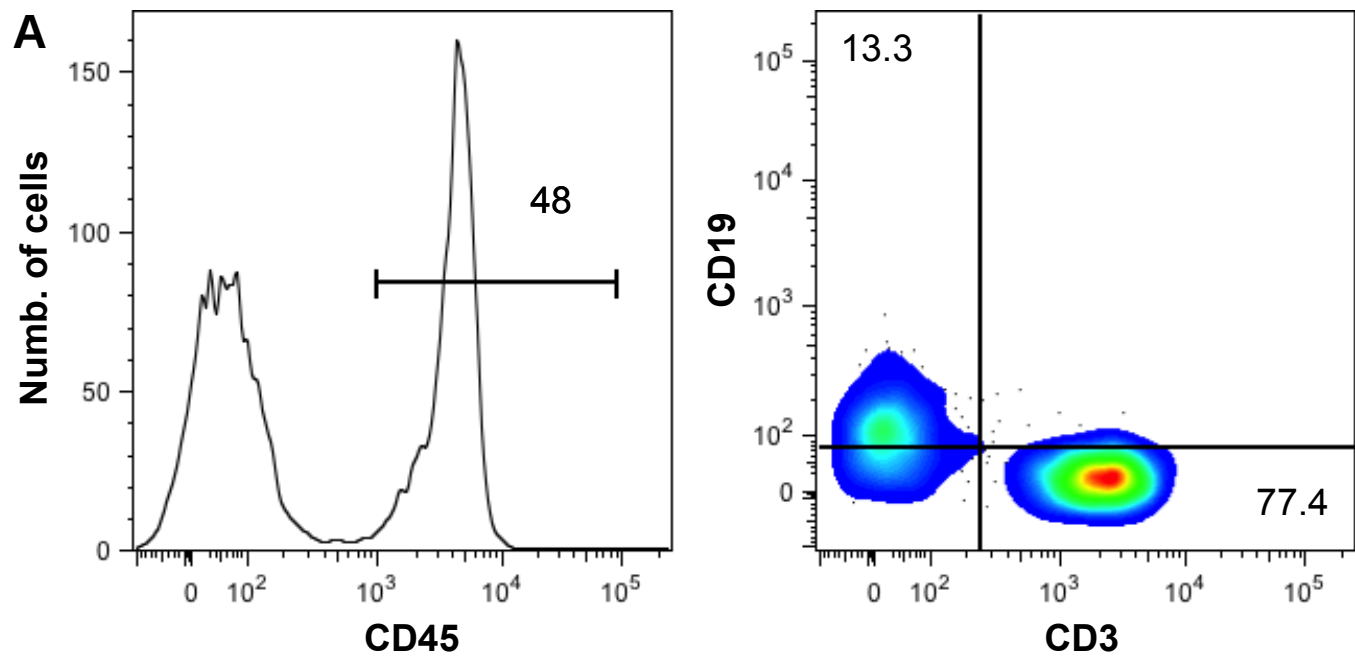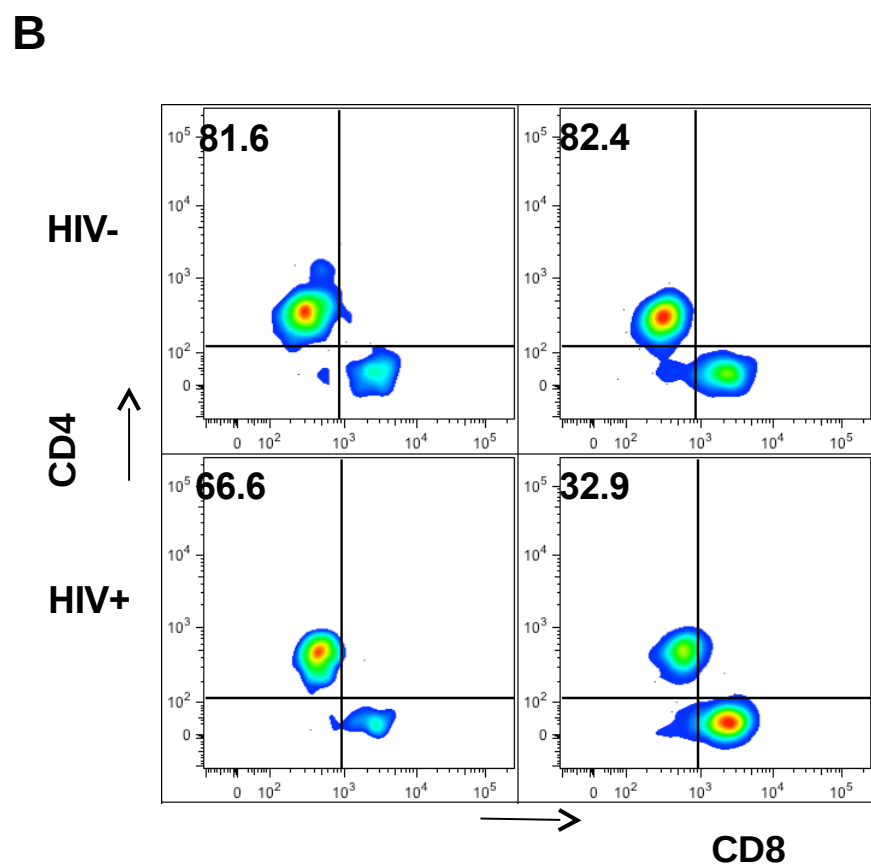

**PBS**

**1 $\mu$ g MT807R1**

**HIT8**

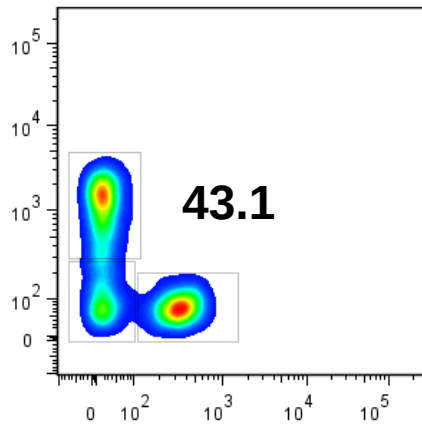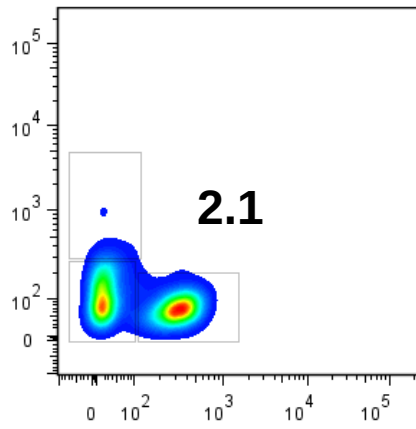

**CD8**

**RPA-T8**

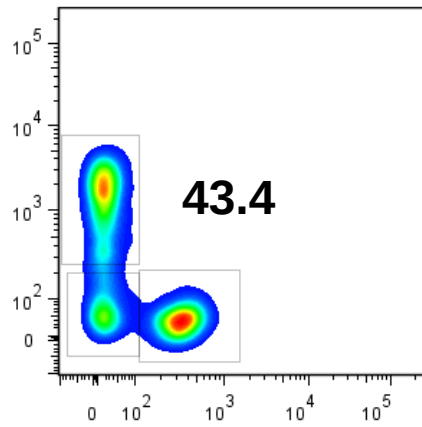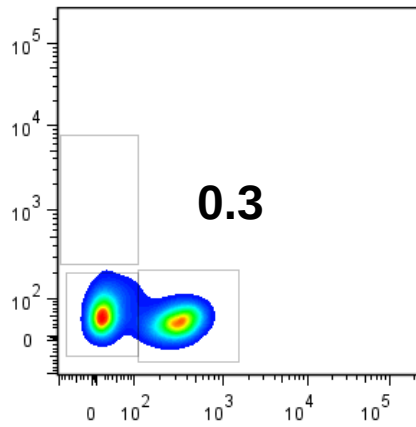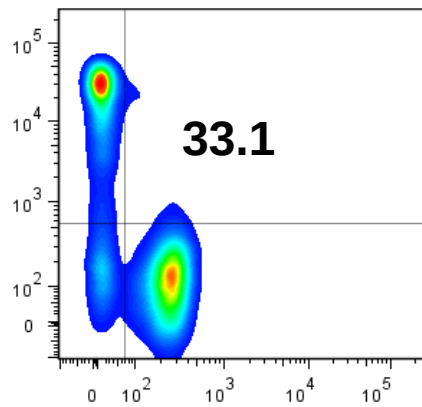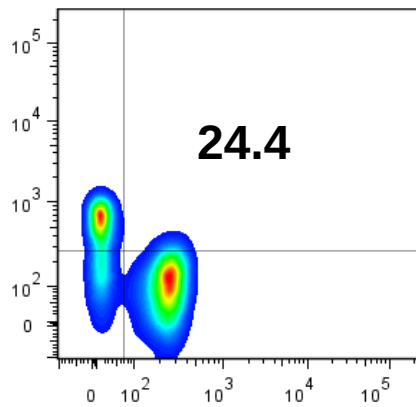

**DK25**

**CD4**

PBS

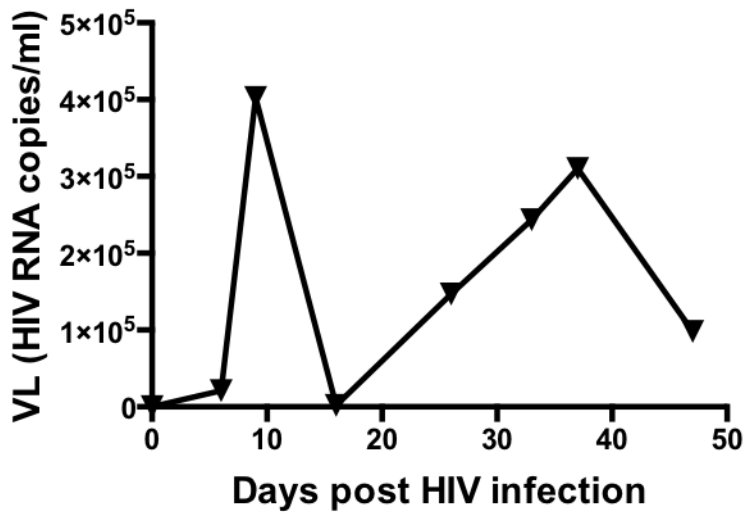

CD8-depleted

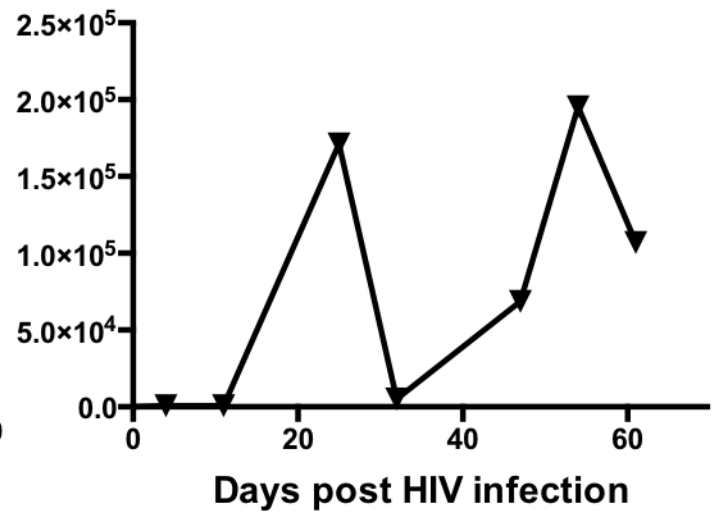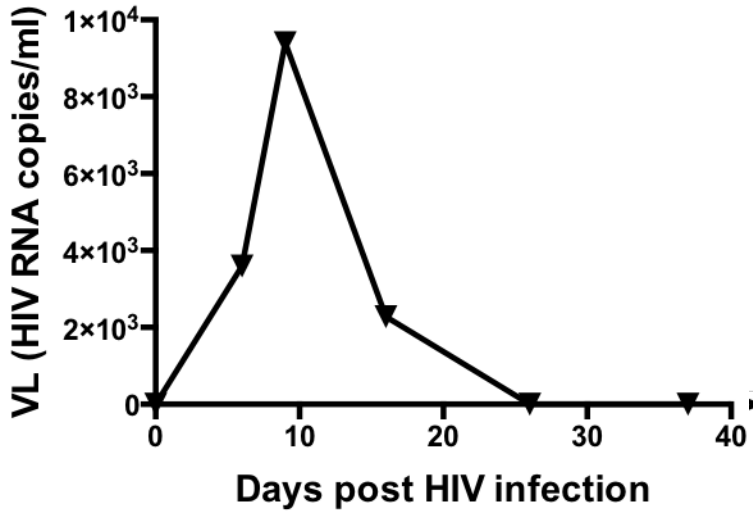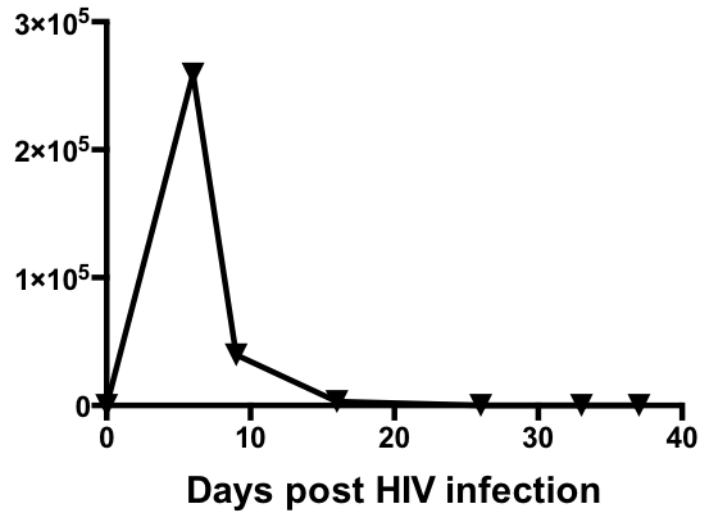

Supplement: S1 Fig — (a) Frequencies of CD3+ T cells and CD19+ B cells in CD45+ human cells was measured in the blood of 10 NSG HuMice from 14 to 20 weeks of age at the indicated days relative to HIV infection (b) Representative CD4/CD8 profiles from non-infected (HIV-) and infected (HIV+) NSG HuMice at the indicated time after infection with 15ng of HIV Bal at 19 weeks of age. (PDF) [file pone.0138420.s001.pdf]

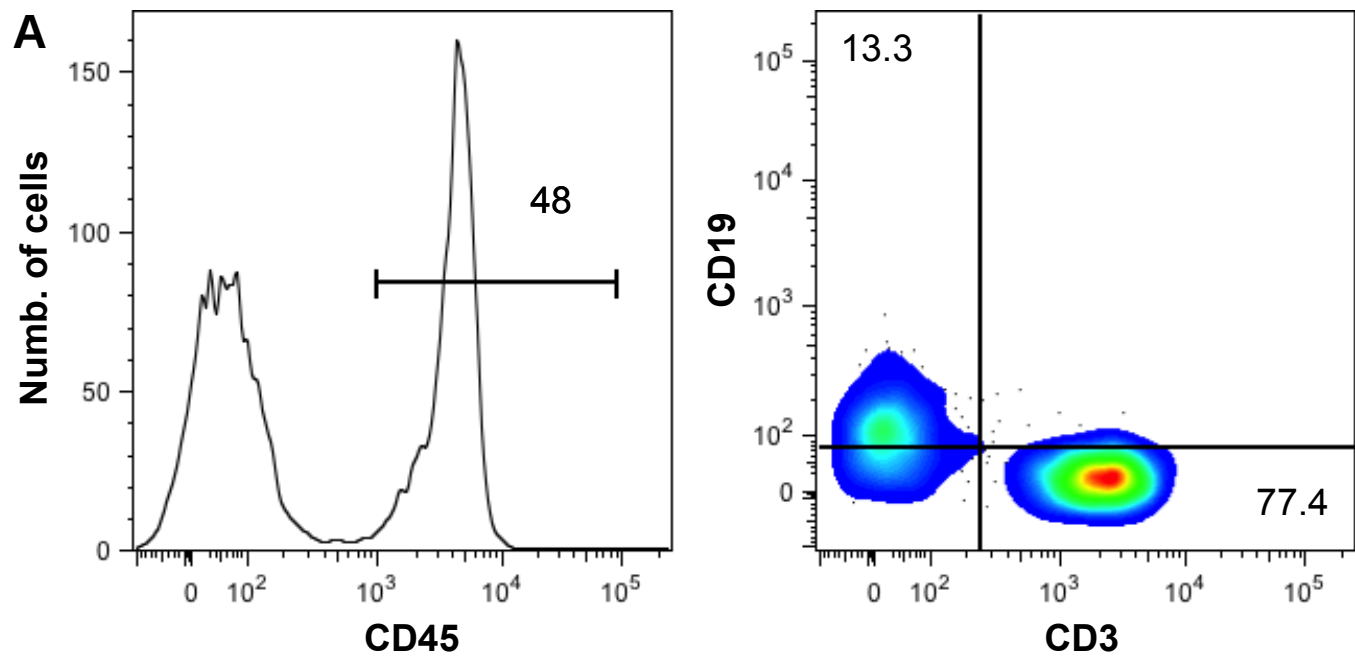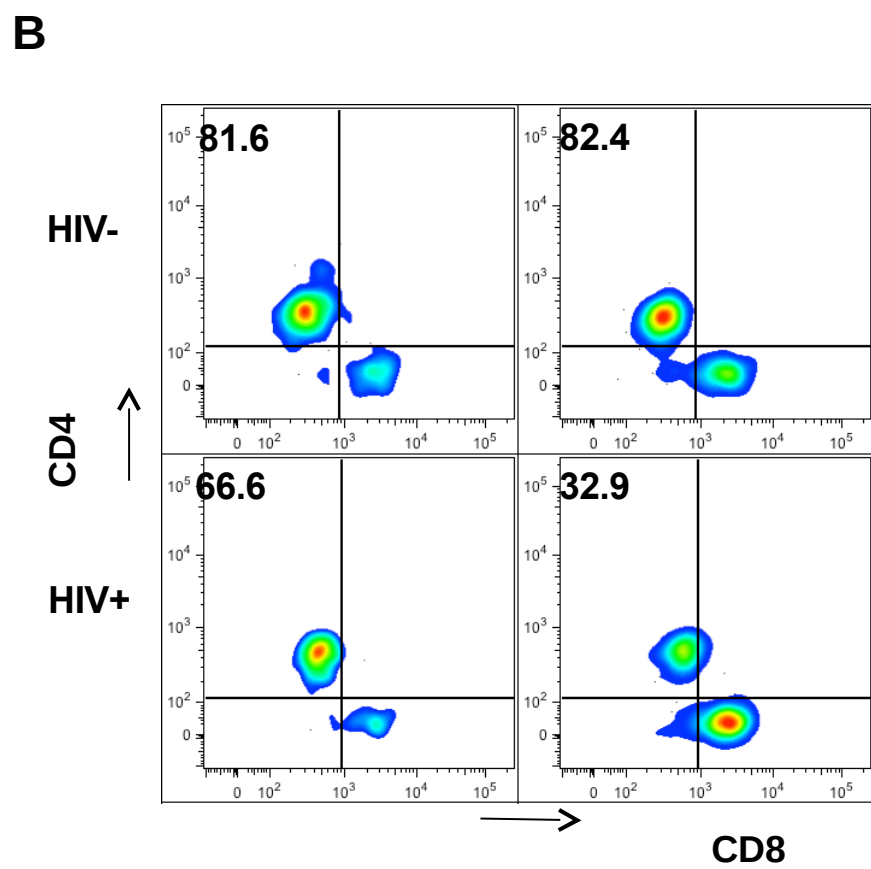

**PBS**

**1 $\mu$ g MT807R1**

**HIT8**

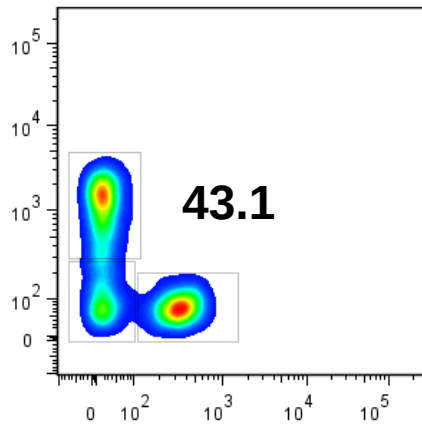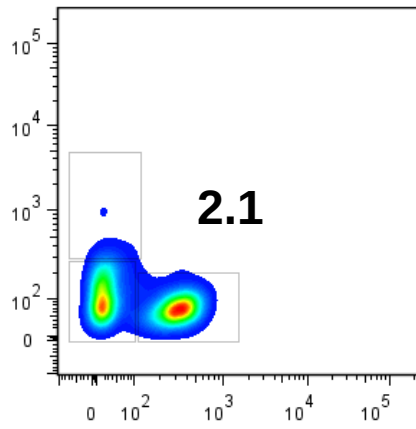

**CD8**

**RPA-T8**

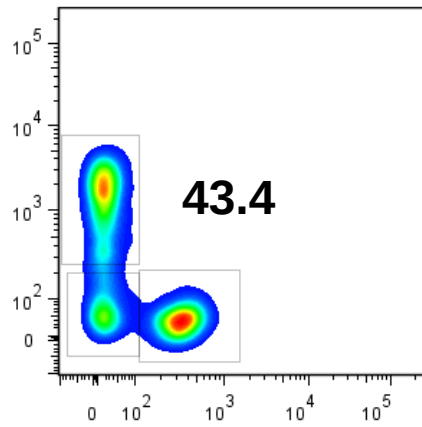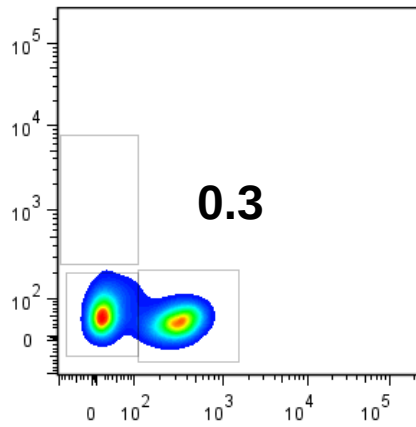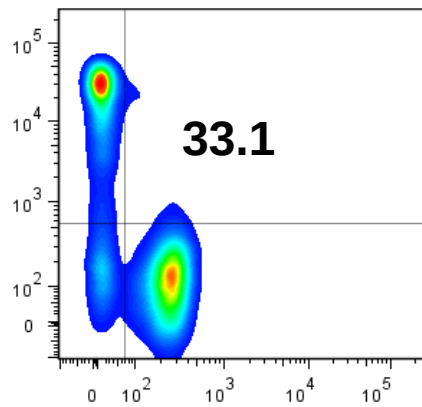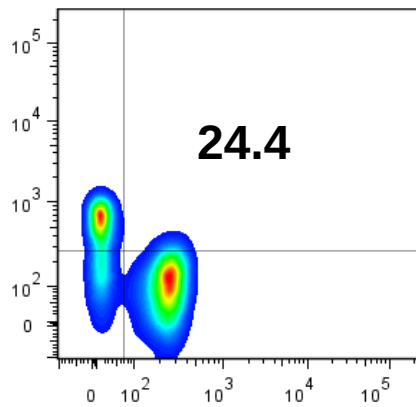

**DK25**

**CD4**

PBS

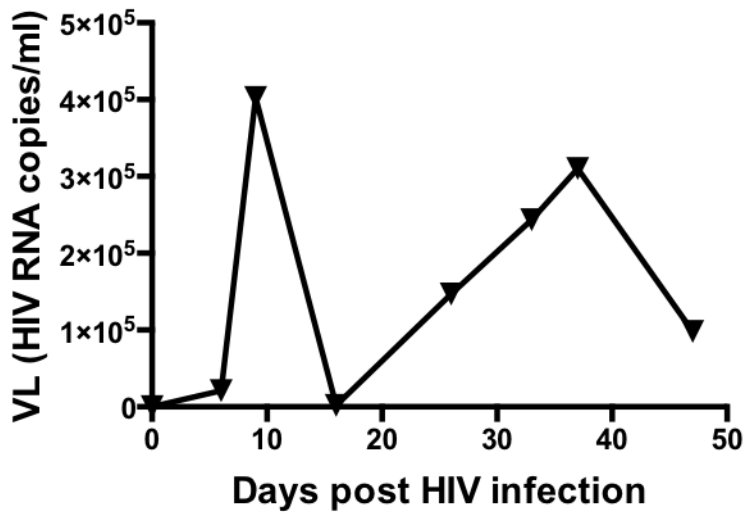

CD8-depleted

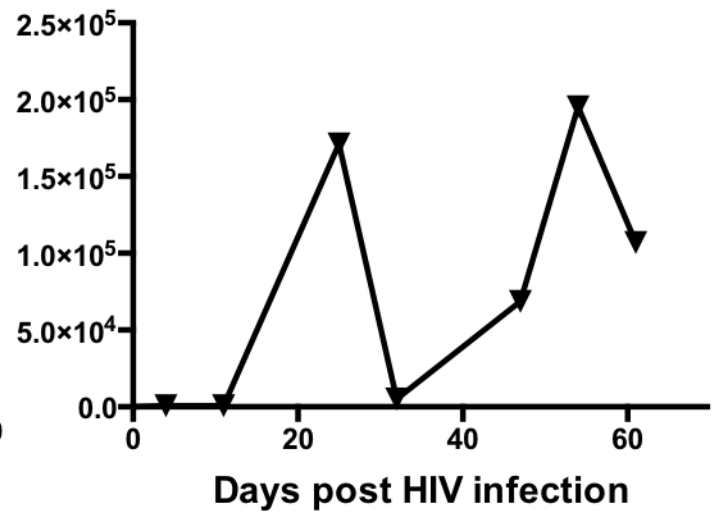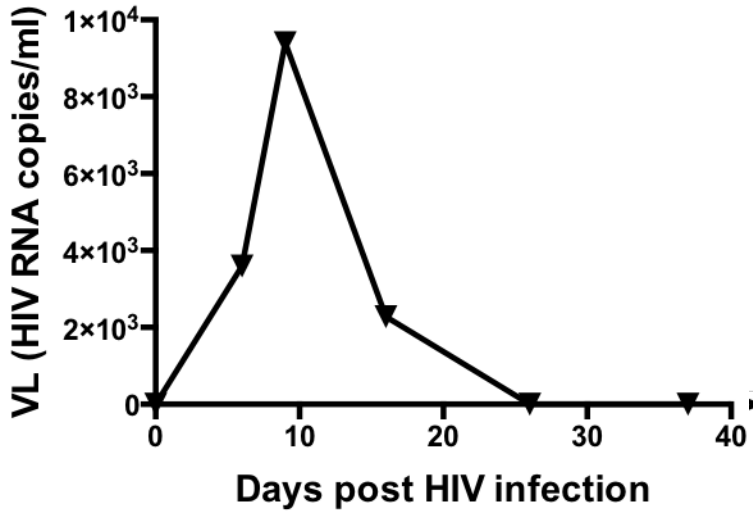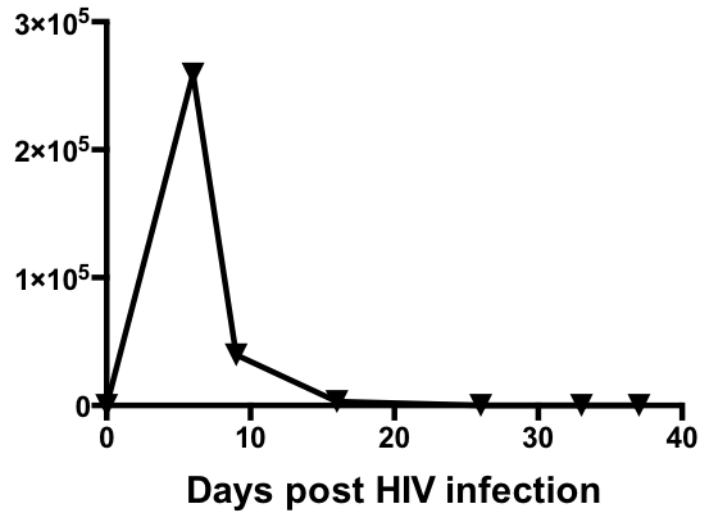

Supplement: S2 Fig — Resting PBMC were incubated 30 min. on ice with PBS or 1μg of MT807R1. Cells were then washed and stained with fluorescent mAbs anti-CD3, anti-CD4 and various anti-CD8 clones as indicated on the figure. Numbers on the profiles indicate the percentage of CD8+ cells. (PDF) [file pone.0138420.s002.pdf]

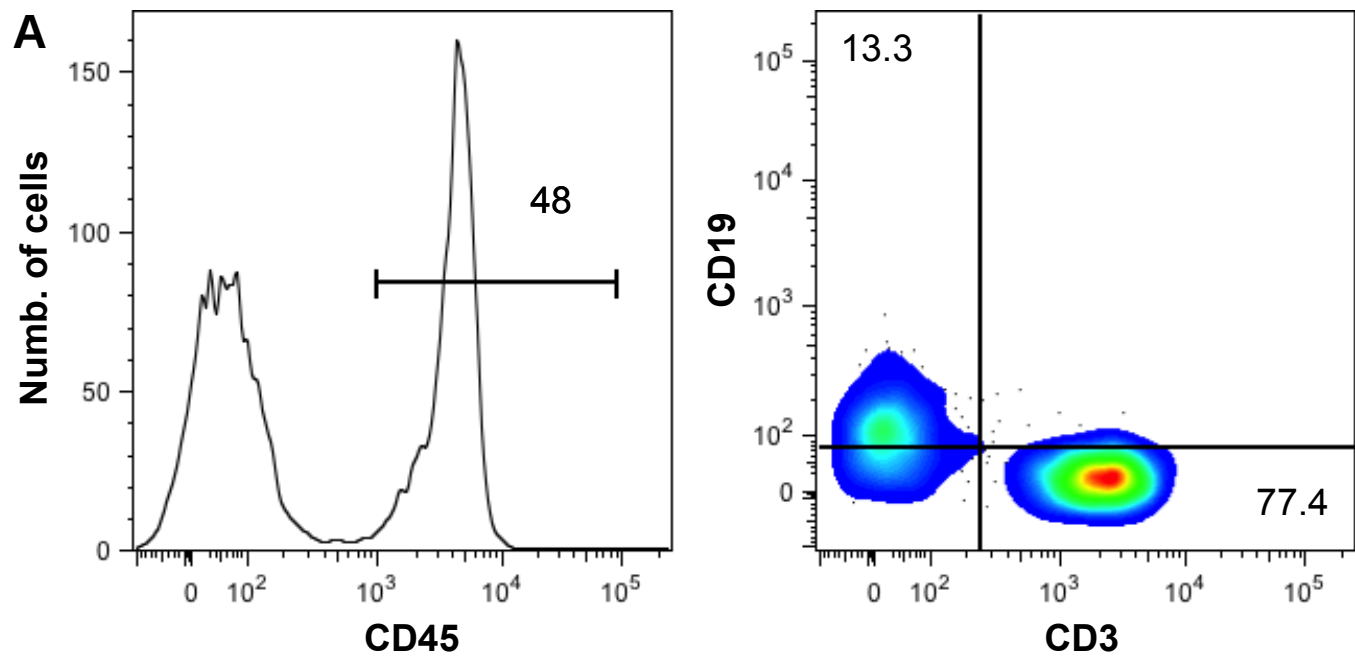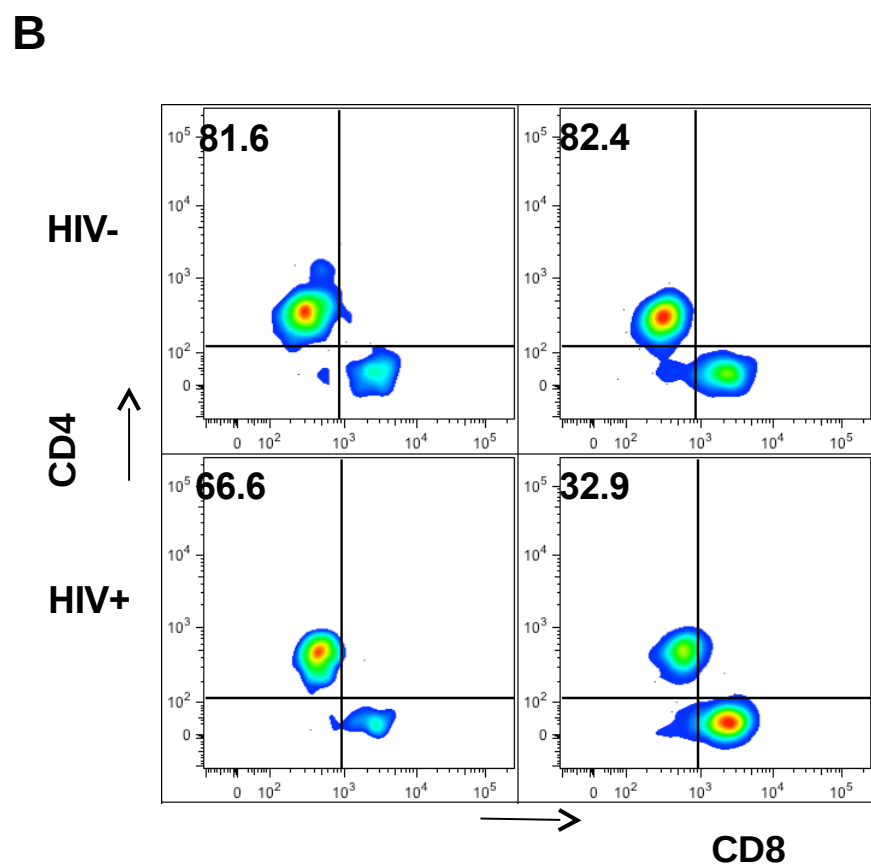

**PBS**

**1 $\mu$ g MT807R1**

**HIT8**

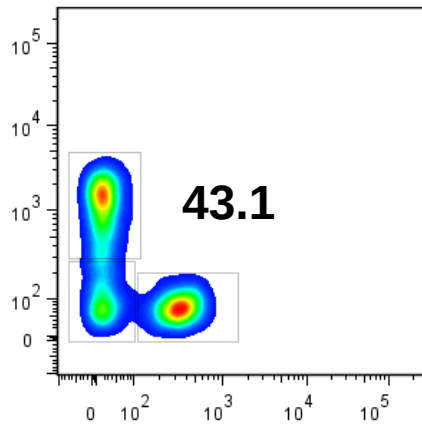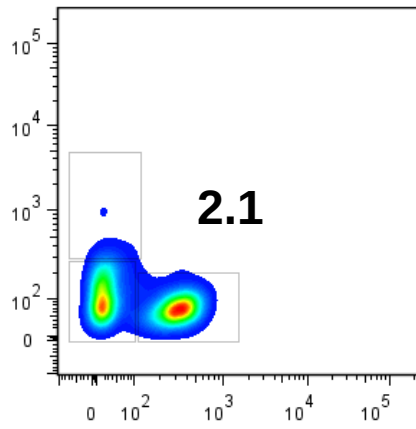

**CD8**

**RPA-T8**

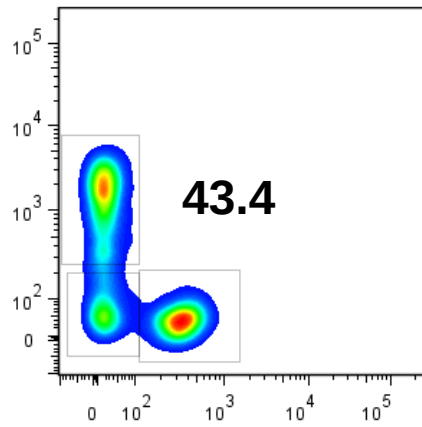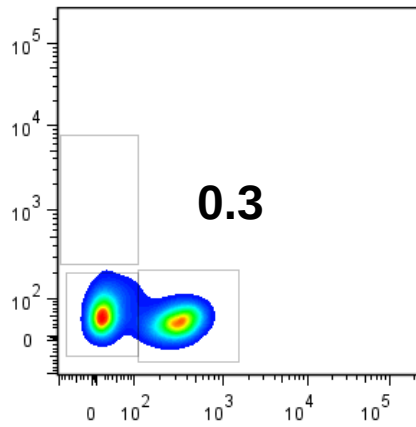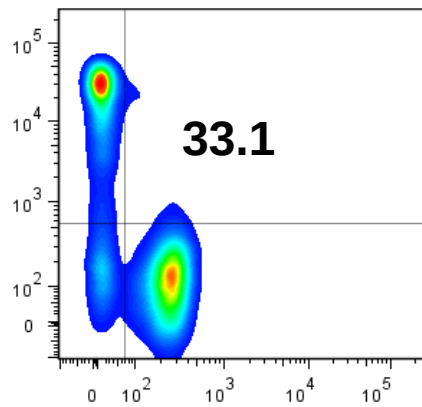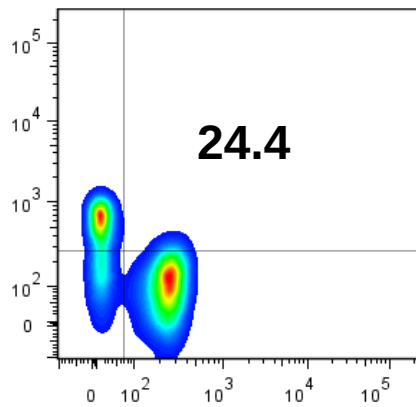

**DK25**

**CD4**

PBS

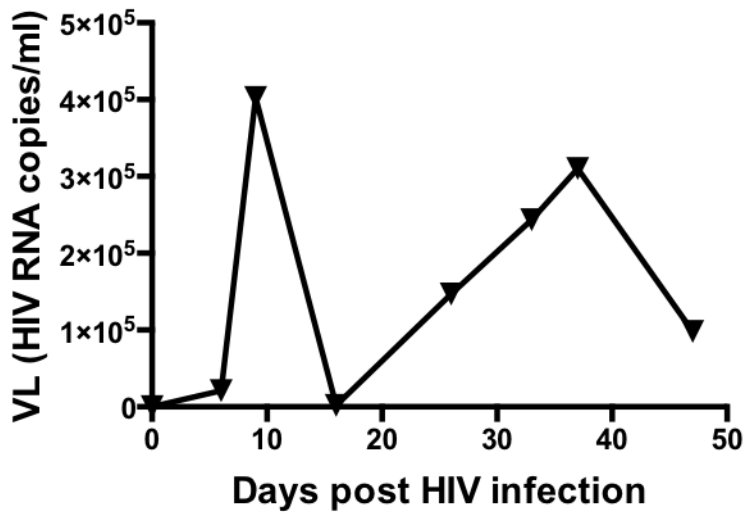

CD8-depleted

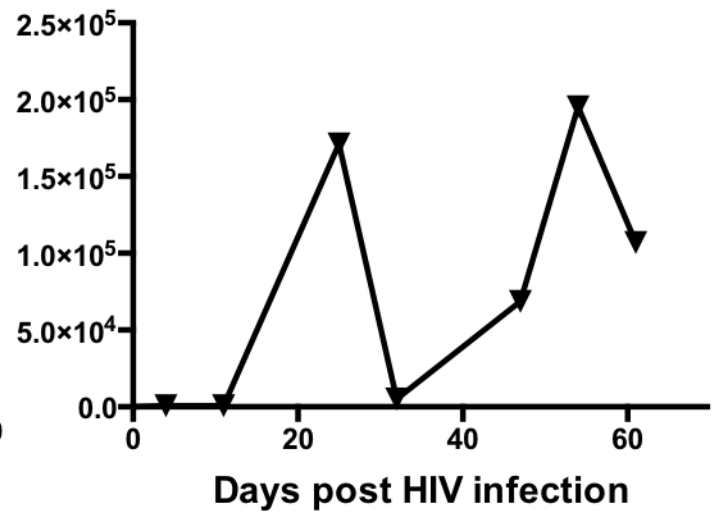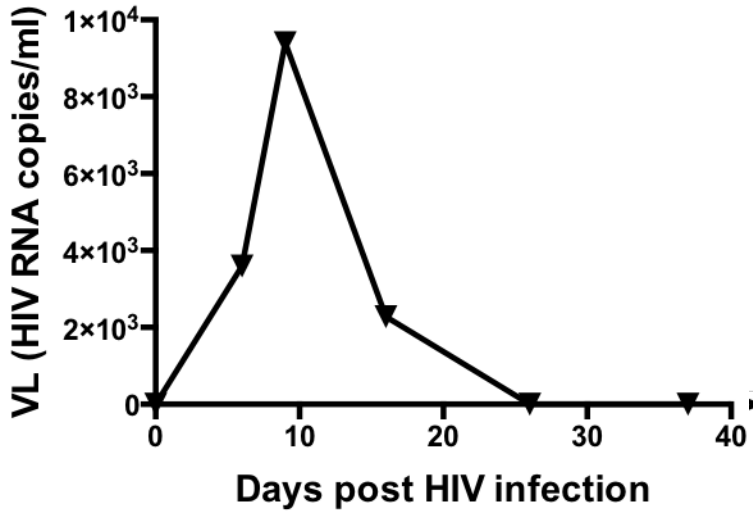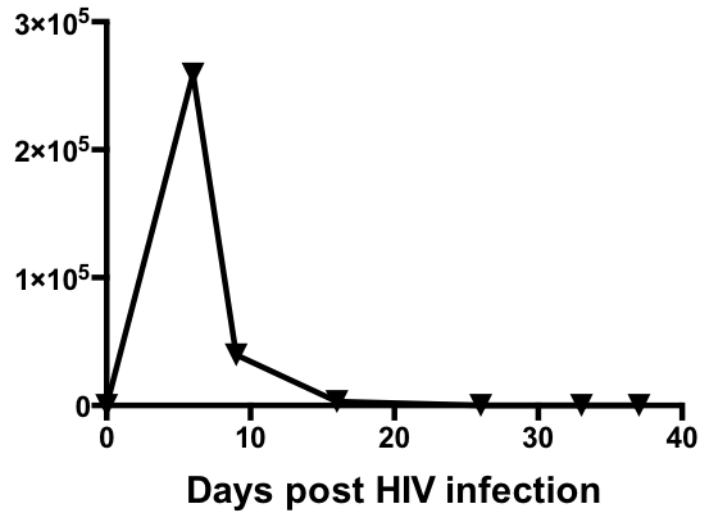

Supplement: S3 Fig — 17 to 36 weeks-old NSG HuMice were injected with PBS or 10mg/kg MT807R1 (CD8-depleted) and infected 3 days later with HIV NLAD8 strain. Viremia was determined using the Cobas Roche amplification PCR. (PDF) [file pone.0138420.s003.pdf]
